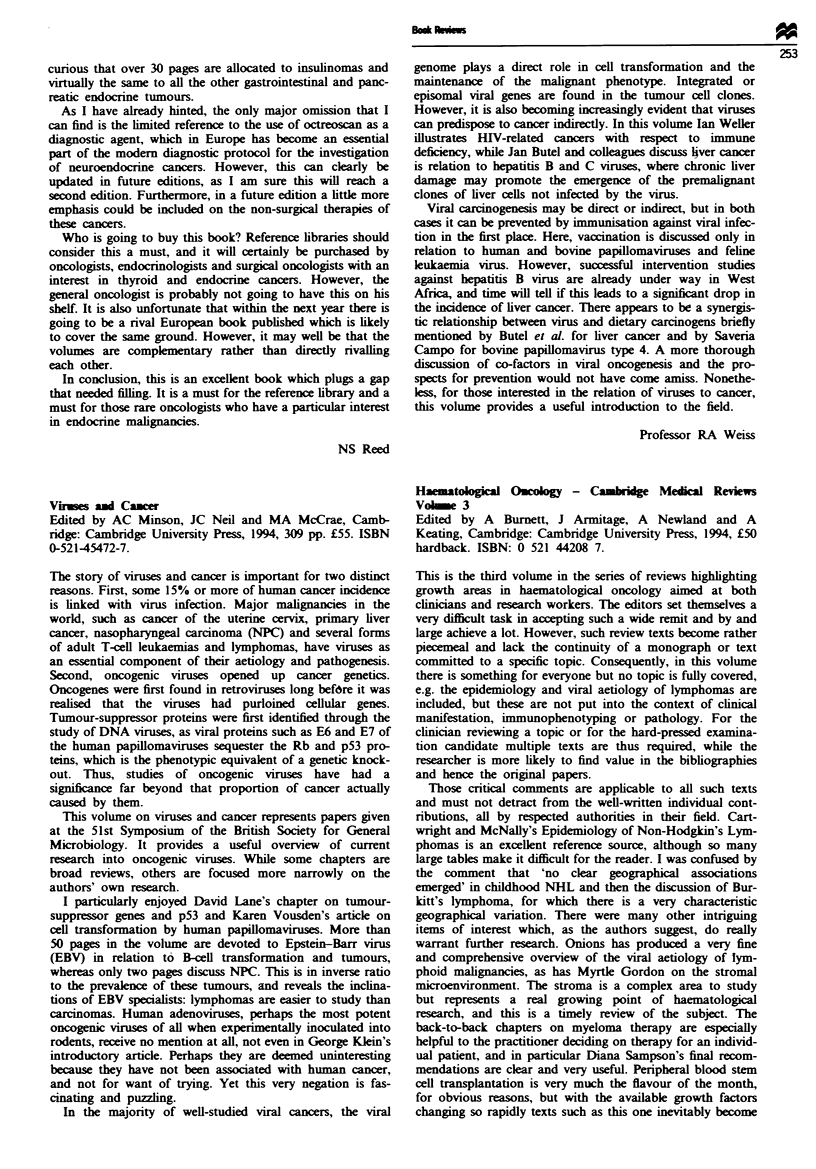# Viruses and Cancer

**Published:** 1995-07

**Authors:** RA Weiss


**Viruses and Cancer**

Edited by AC Minson, JC Neil and MA McCrae, Cambridge: Cambridge University Press, 1994, 309 pp. £55. ISBN 0-521-45472-7.

The story of viruses and cancer is important for two distinct reasons. First, some 15% or more of human cancer incidence is linked with virus infection. Major malignancies in the world, such as cancer of the uterine cervix, primary liver cancer, nasopharyngeal carcinoma (NPC) and several forms of adult T-cell leukaemias and lymphomas, have viruses as an essential component of their aetiology and pathogenesis. Second, oncogenic viruses opened up cancer genetics. Oncogenes were first found in retroviruses long before it was realised that the viruses had purloined cellular genes. Tumour-suppressor proteins were first identified through the study of DNA viruses, as viral proteins such as E6 and E7 of the human papillomaviruses sequester the Rb and p53 proteins, which is the phenotypic equivalent of a genetic knockout. Thus, studies of oncogenic viruses have had a significance far beyond that proportion of cancer actually caused by them.

This volume on viruses and cancer represents papers given at the 51st Symposium of the British Society for General Microbiology. It provides a useful overview of current research into oncogenic viruses. While some chapters are broad reviews, others are focused more narrowly on the authors' own research.

I particularly enjoyed David Lane's chapter on tumour-suppressor genes and p53 and Karen Vousden's article on cell transformation by human papillomaviruses. More than 50 pages in the volume are devoted to Epstein–Barr virus (EBV) in relation to B-cell transformation and tumours, whereas only two pages discuss NPC. This is in inverse ratio to the prevalence of these tumours, and reveals the inclinations of EBV specialists: lymphomas are easier to study than carcinomas. Human adenoviruses, perhaps the most potent oncogenic viruses of all when experimentally inoculated into rodents, receive no mention at all, not even in George Klein's introductory article. Perhaps they are deemed uninteresting because they have not been associated with human cancer, and not for want of trying. Yet this very negation is fascinating and puzzling.

In the majority of well-studied viral cancers, the viral genome plays a direct role in cell transformation and the maintenance of the malignant phenotype. Integrated or episomal viral genes are found in the tumour cell clones. However, it is also becoming increasingly evident that viruses can predispose to cancer indirectly. In this volume Ian Weller illustrates HIV-related cancers with respect to immune deficiency, while Jan Butel and colleagues discuss liver cancer is relation to hepatitis B and C viruses, where chronic liver damage may promote the emergence of the premalignant clones of liver cells not infected by the virus.

Viral carcinogenesis may be direct or indirect, but in both cases it can be prevented by immunisation against viral infection in the first place. Here, vaccination is discussed only in relation to human and bovine papillomaviruses and feline leukaemia virus. However, successful intervention studies against hepatitis B virus are already under way in West Africa, and time will tell if this leads to a significant drop in the incidence of liver cancer. There appears to be a synergistic relationship between virus and dietary carcinogens briefly mentioned by Butel *et al.* for liver cancer and by Saveria Campo for bovine papillomavirus type 4. A more thorough discussion of co-factors in viral oncogenesis and the prospects for prevention would not have come amiss. Nonetheless, for those interested in the relation of viruses to cancer, this volume provides a useful introduction to the field.

Professor RA Weiss